# Unexplained facts about subcutaneous emphysema after cleft lip surgery

**DOI:** 10.4103/0019-5049.76570

**Published:** 2011

**Authors:** Anju Ghai, Raman Wadhera, Sanjay Johar, Nidhi Garg

**Affiliations:** Department of Anaesthesiology and Critical Care, PGIMS, Rohtak, Haryana, India

Sir,

We read with interest the article titled “ A case of severe subcutaneous emphysema in the postoperative period following cleft lip surgery” by Vijayakumar and colleagues in the March-April 2010 issue.[1] We appreciate the authors for managing the patient successfully. The authors have attributed the emphysema to the retching and vomiting which could have increased the alveolar pressure leading to alveolar rupture. A few facts remained unexplained in the article. The Valsalva manoeuvre during retching can certainly cause a rise in alveolar pressure but is that rise sufficient enough to cause alveolar rupture? We could not find this fact in the literature. If it can lead to alveolar rupture, can that cause breach in alveoli leading to extensive subcutaneous emphysema also needs explanation. The latent period was too long. The vomiting episodes occurred in the immediate postoperative period while the emphysema developed after 6 h. The oesophagogram was done to rule out oesophageal rupture. Did the authors anticipate oesophageal rupture due to vomiting? Oesophageal rupture normally does not lead to subcutaneous emphysema. This also needs explanation. Four cases of subcutaneous emphysema and pneumomediastinum with secondary pneumothoraces have been reported after self-induced punctures in the oral cavity.[2] It constitutes an uncommon entity. This can as well explain the subcutaneous emphysema after cleft lip surgery as mentioned by authors.

We also encountered a similar patient of subcutaneous emphysema which developed after rigid bronchoscopy and it increased to the extent that it encircled the whole of the neck and face. The child became dyspnoeic but was managed with oxygen and nebulisation. Serial chest X-rays show resolution of emphysema. The cause could be clearly defined in our patient while the author’s case needs explanation.
